# Supplementary material for: Synthesis and Optical Properties of a Novel Hybrid Nanosystem Based on Covalently Modified nSiO2 Nanoparticles with a Curcuminoid Molecule
Source: Nanomaterials (Basel). 2024 Jun 13;14(12):1022. doi: 10.3390/nano14121022 (PMC11207103; doi:10.3390/nano14121022)
Supplement: Supplementary file 1 [file nanomaterials-14-01022-s001.zip › nanomaterials-2987402-supplementary.pdf]

Supplementary Materials

Synthesis and Optical Properties of a Novel Hybrid Nanosystem Based on Covalently Modified $nSiO_2$ Nanoparticles with a Curcuminoid Molecule

Nicole Parra-Muñoz [1,2,*], Valentina López-Monsalves [1,3], Rodrigo Espinoza-González [1], Daniel Aravena [4], Nancy Pizarro [5] and Monica Soler [1,*]

[1] Department of Chemical Engineering, Biotechnology and Materials, Faculty of Physical and Mathematical Sciences, Universidad de Chile, Santiago 8370456, Chile; roespino@ing.uchile.cl (R.E.-G.)
[2] Centro de Materiales para la Transición y Sostenibilidad Energética, Comisión Chilena de Energía Nuclear, Ruta 68, km 20, 9020000, Pudahuel, Santiago 7600713, Chile
[3] Departamento de Química Farmacológica y Toxicológica, Facultad de Ciencias Químicas y Farmacéuticas, Universidad de Chile, Santiago 8380494, Chile
[4] Departamento de Química de los Materiales, Facultad de Química y Biología, Universidad de Santiago de Chile (USACH), Casilla 40, Correo 33, Santiago 9170002, Chile; daniel.aravena.p@usach.cl
[5] Departamento de Ciencias Químicas, Facultad de Ciencias Exactas, Universidad Andrés Bello, Viña del Mar 2520000, Chile; npizarro@unab.cl
* Correspondence: nicole.parra@cchen.cl (N.P.-M.); msoler@ing.uchile.cl (M.S.)

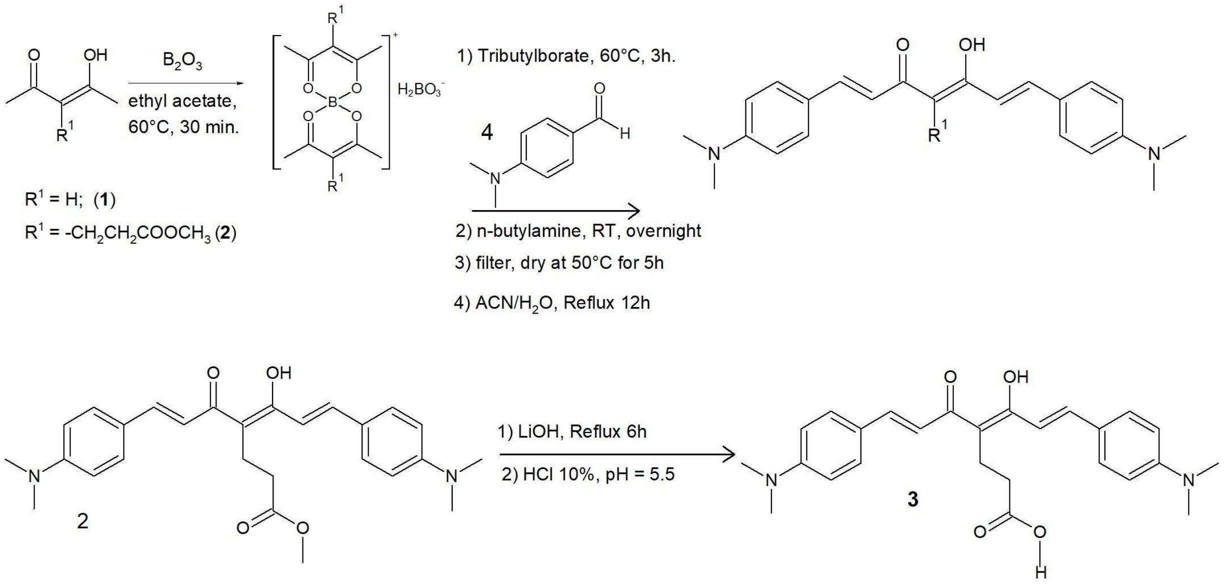

Scheme S1. Synthesis pathway of curcuminoid molecules **1**, **2** and **3**

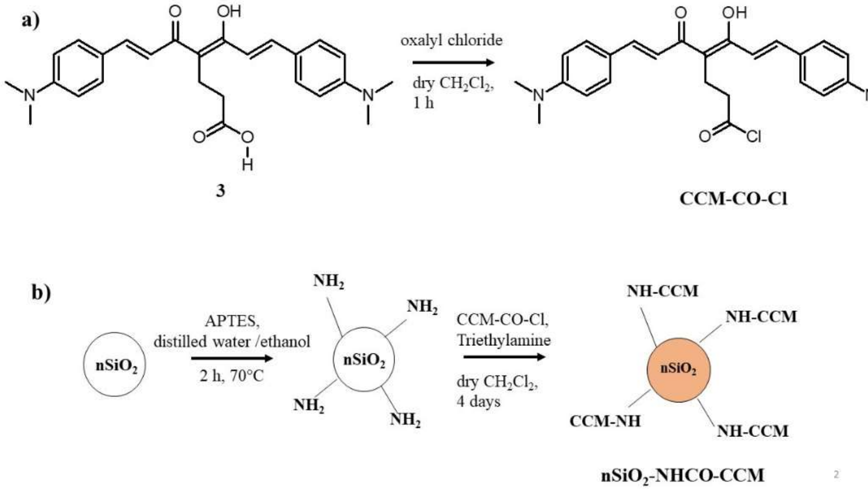

Scheme S2. Synthesis pathway of $nSiO_2$-NHCO-CCM

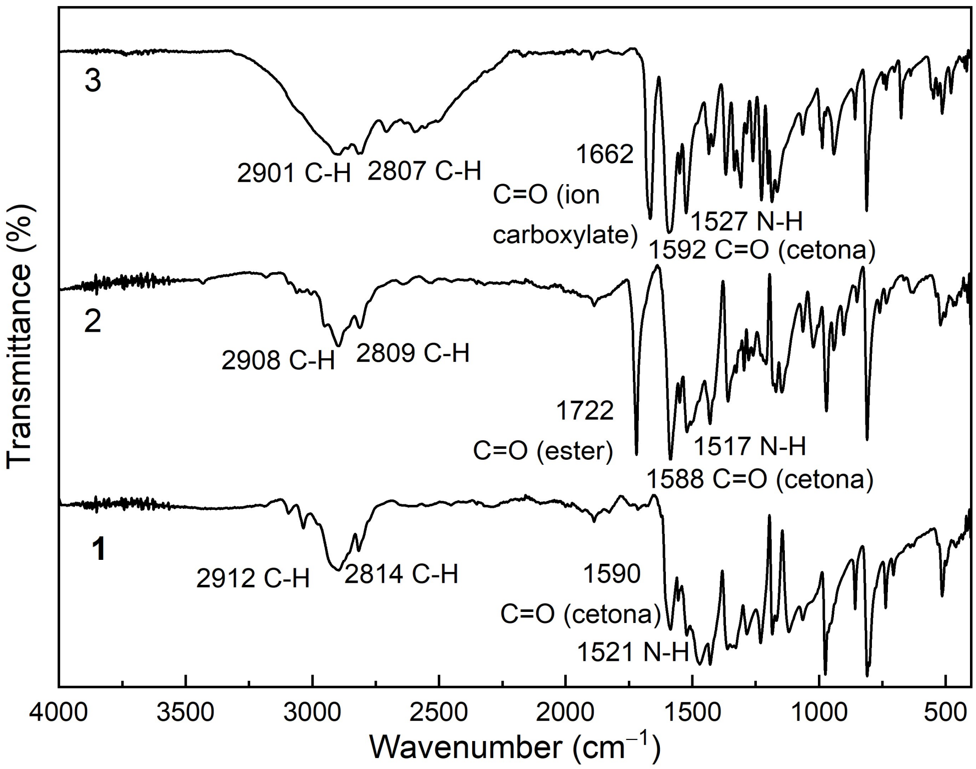

Figure S1. FTIR-ATR spectrum of curcuminoid molecules.

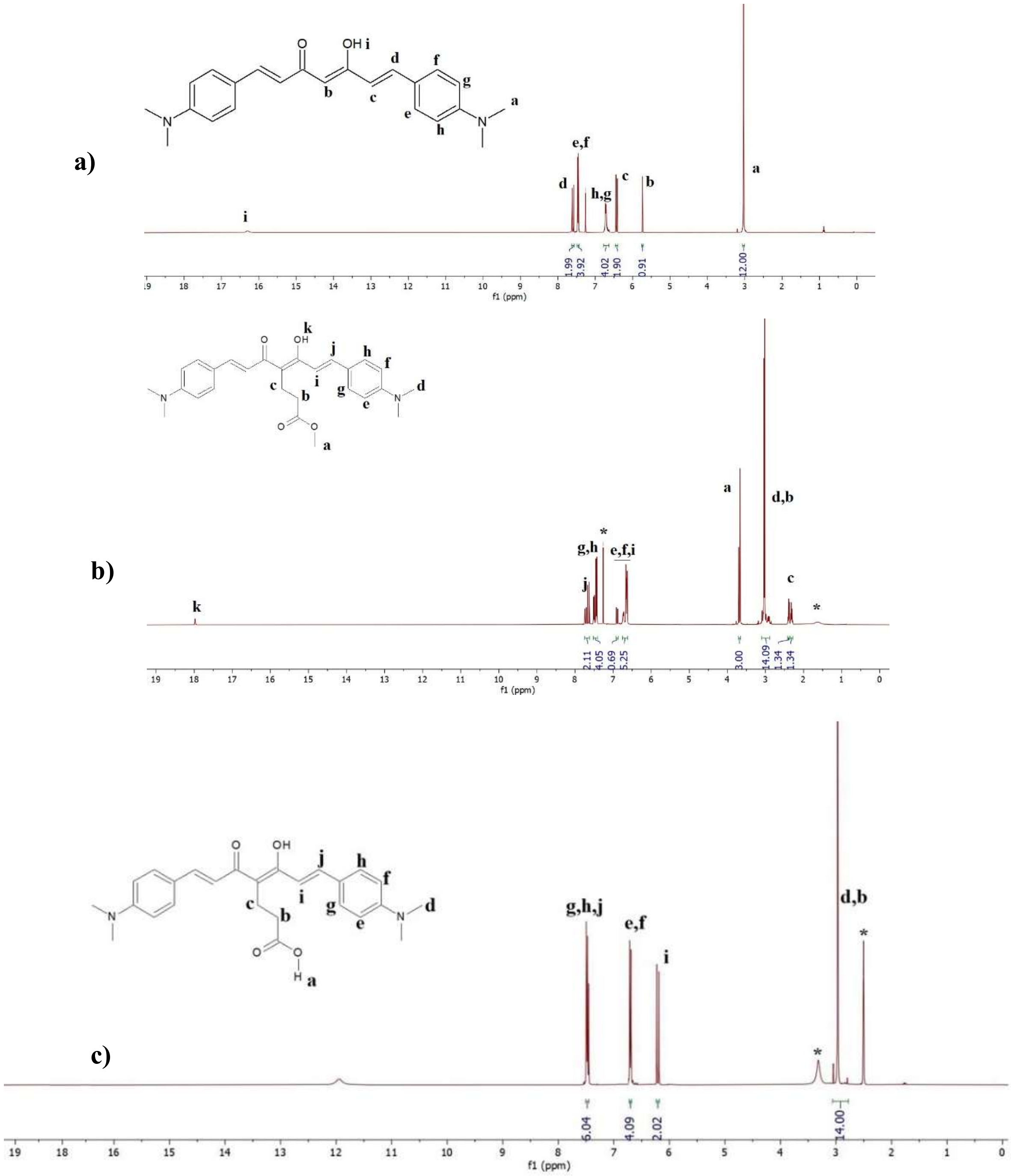

Figure S2. Nuclear magnetic resonance spectrum, ^{1}H-RMN, of curcuminoid molecules in $CDCl_3$. a) curcuminoid **1**; b) curcuminoid **2**; c) curcuminoid **3.**

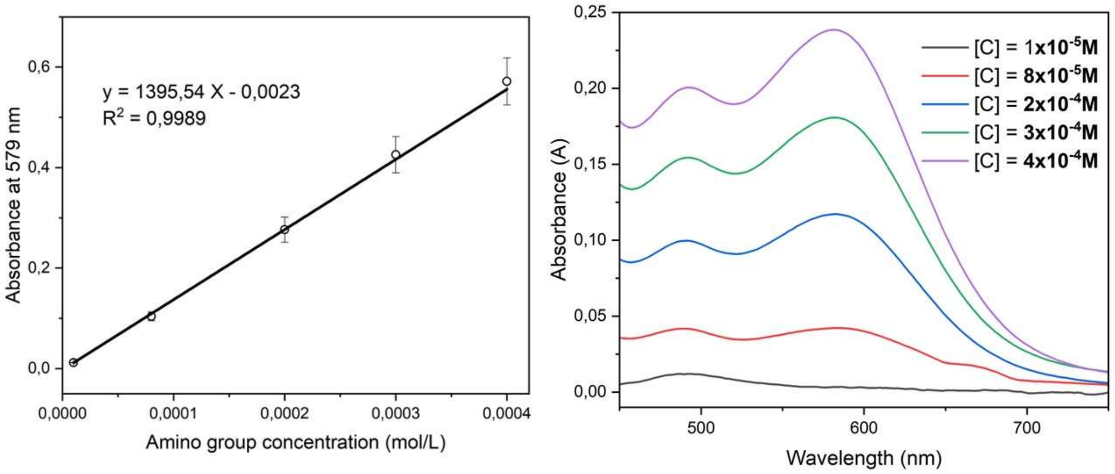

Figure S3. a) Calibration curve, b) absorption spectra of the different butylamine solutions carried out employing the Kaiser test.

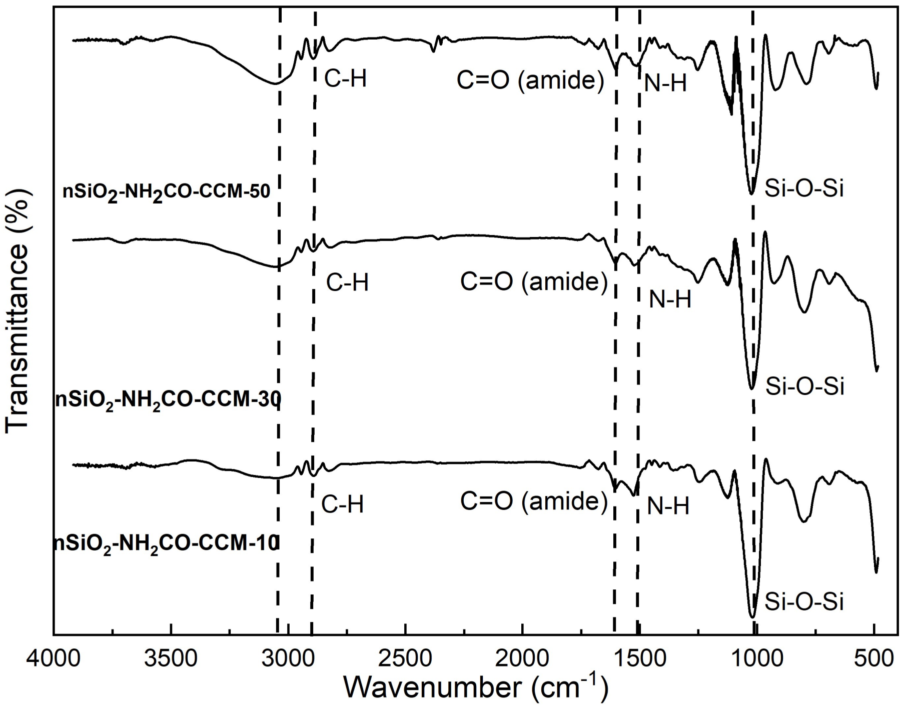

Figure S4: FTIR-ATR spectrum of nanoparticles functionalized with curcuminoids.

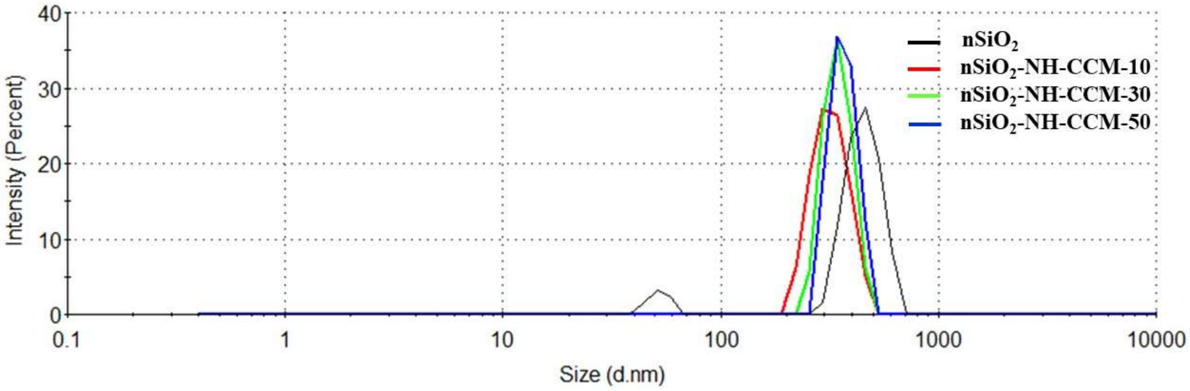

Figure S5. Hydrodynamic diameter distribution of nanoparticles grafted with a curcuminoid molecule.

Table S1. Average hydrodynamic diameter and polydispersity index of the nanoparticles grafted with a curcuminoid molecule.

**nanostructure**	**Z average (nm) ± SD (nm)**	**%RDS**	**PDI (polydispersity index)**
nSiO$_2$	518 ± 62.7	7.84	0.511 ± 0.03
nSiO$_2$-NH-CCM-10	388 ± 11.2	2.90	0.113 ± 0.03
nSiO$_2$-NH-CCM-30	459 ± 22.8	6.11	0.127 ± 0.03
nSiO$_2$-NH-CCM-50	499 ± 16.9	3.40	0.150 ± 0.10

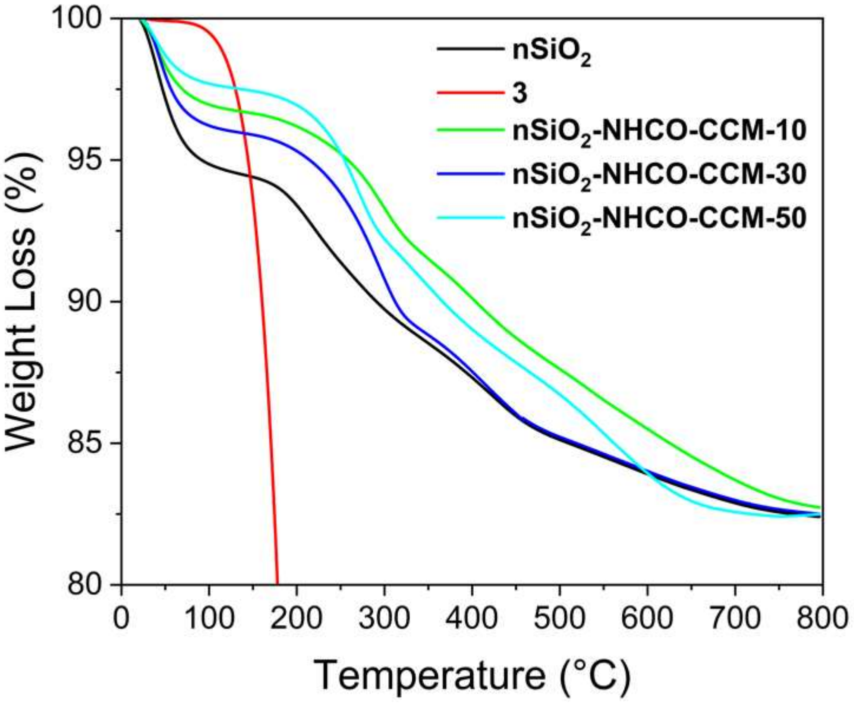

Figure S6. TGA thermograms of curcuminoid molecule **3** and nanoparticles grafted with a curcuminoid molecule.

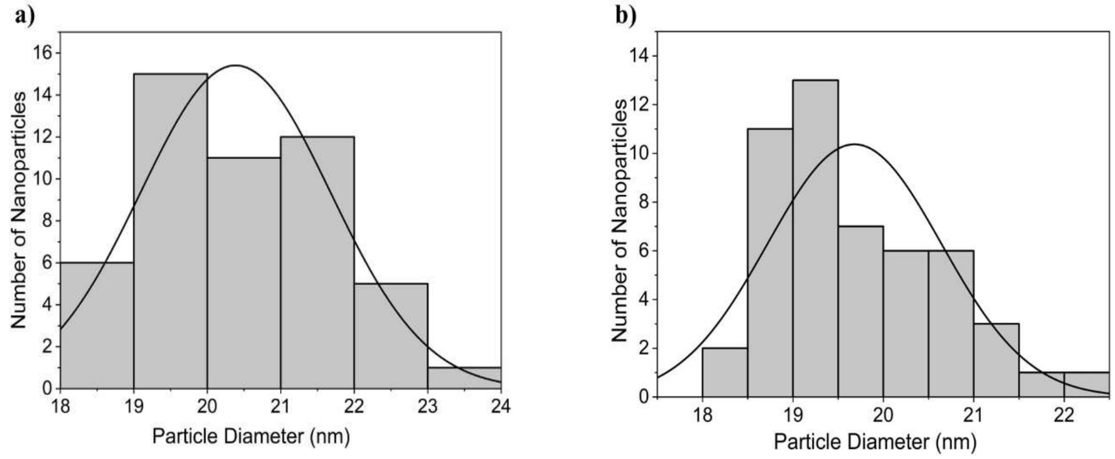

Figure S7. Histograms of the TEM images for a) $nSiO_2$ and b) $nSiO_2$-NHCO-CCM-30.

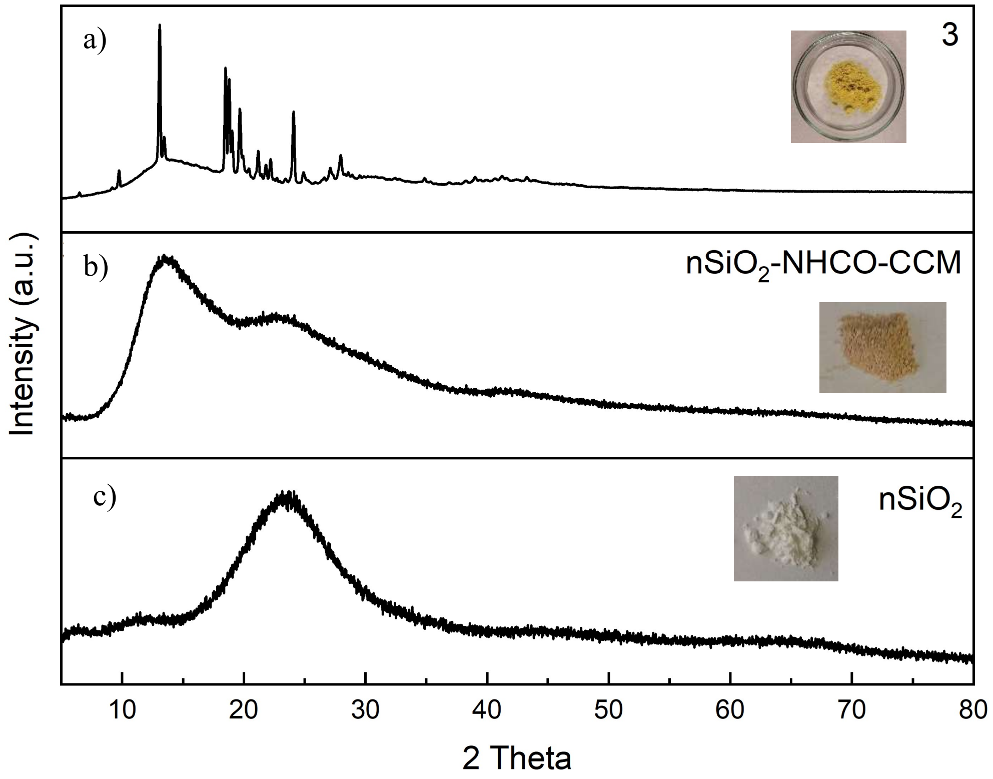

Figure S8. DRX diffractograms of a) curcuminoid **3**, b) $nSiO_2$-NHCO-CCM, and c) $nSiO_2$.

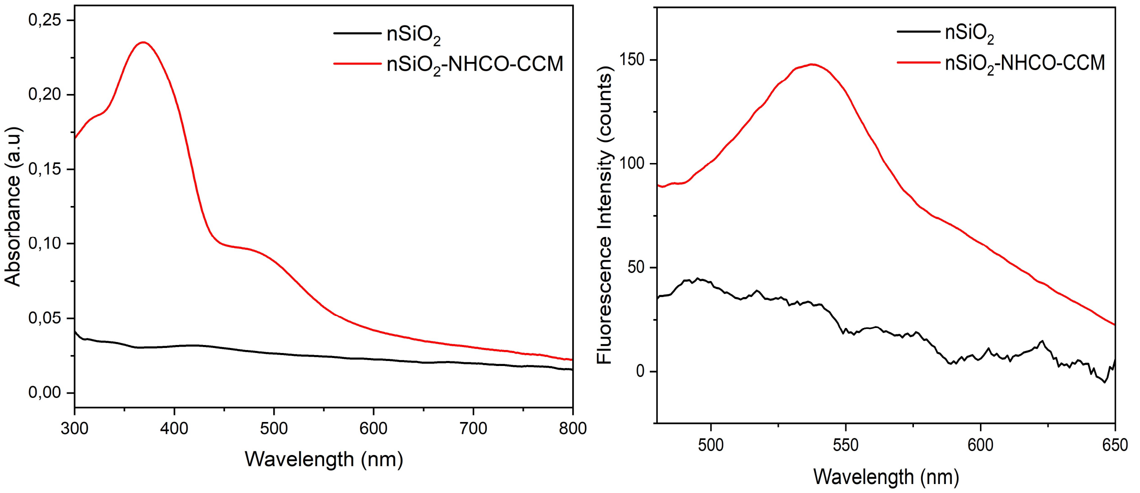

Figure S9. Absorption (a) and emission spectra (b) of $nSiO_2$ and $nSiO_2$-NHCO-CCM suspension in CH_2Cl_2.

Table S2. Photophysical data of curminoids **1**, **2** and **3** and $nSiO_2$-NHCO-CCM in CH_2Cl_2 solution

**Solvents**	**1**			**2**			**3**			**$nSiO_2$-NHCO-CCM**		
	**λmax**	**λemi**	**Δυstokes (cm^{-1})**	**λmax**	**λemi**	**Δυstokes (cm^{-1})**	**λmax**	**λemi**	**Δυstokes (cm^{-1})**	**λmax**	**λemi**	**Δυstokes (cm^{-1})**
**Hexane**	444	486	1946	458	502	1913	346	390	3261	468	517	2025
**Cyclohexane**	448	491	2232	462	506	1882	344	392	3560	473	521	1948
**Dioxane**	468	541	2883	479	546	2562	355	413	3956	466	519	2191
**Toluene**	468	525	2320	480	537	2211	358	422	4236	467	531	2581
**Chloroform**	487	561	2709	498	572	2598	363	440	4821	468	535	2676
**Ethyl acetate**	469	543	2906	479	548	2629	353	424	4744	469	537	2670
**Tetrahydrofuran**	473	544	2759	482	549	2532	355	420	4359	466	535	2768
**Dichloromethane**	488	565	2793	500	573	2548	363	445	5076	472	538	2599
**Acetone**	483	561	2879	493	567	2647	357	443	5438	468	531	2490
**Ethanol**	487	609	4114	501	610	3567	358	452	5809	467	531	2581
**Acetonitrile**	486	587	3540	498	596	3302	361	451	5528	462	535	2953
**DMF**	488	590	3543	509	597	2896	359	457	5973	459	540	3234
**DMSO**	502	606	3419	518	601	2666	363	451	5376	453	540	3522

Table S3. The physical parameters of the solvents

Solvents	η	ε	Kamlet –Taft[1]			Catalán[2]			
			α	β	π*	SA	SB	SP	SdP
Hexane	1.3749	1.88	0.000	0.000	-0.080	0.000	0.056	0.000	0.616
Cyclohexane	1.4262	2.02	0.000	0.000	0.000	0.000	0.073	0.000	0.683
Dioxane	1.4224	2.56	0.370	0.000	0.550	0.000	0.444	0.312	0.737
Toluene	1.4969	2.38	0.110	0.000	0.540	0.000	0.128	0.284	0.782
Chloroform	1.4459	4.89	0.000	0.000	0.580	0.047	0.071	0.614	0.783
Ethyl acetate	1.3724	6.02	0.450	0.000	0.550	0.000	0.542	0.603	0.656
Tetrahydrofuran	1.4072	7.58	0.550	0.000	0.580	0.000	0.591	0.643	0.714
Dichloromethane	1.4242	8.93	0.000	0.300	0.820	0.040	0.178	0.769	0.761
Acetone	1.3587	20.56	0.480	0.000	0.710	0.000	0.475	0.907	0.651
Ethanol	1.3614	24.55	0.770	0.830	0.540	0.400	0.658	0.783	0.633
Acetonitrile	1.3441	35.94	0.310	0.190	0.750	0.044	0.286	0.974	0.645
Dimethylformamide	1.4305	36.71	0.690	0.000	0.880	0.031	0.613	0.977	0.759
Dimethylsulfoxide	1.4393	46.45	0.760	0.000	1.000	0.072	0.647	1.000	0.830

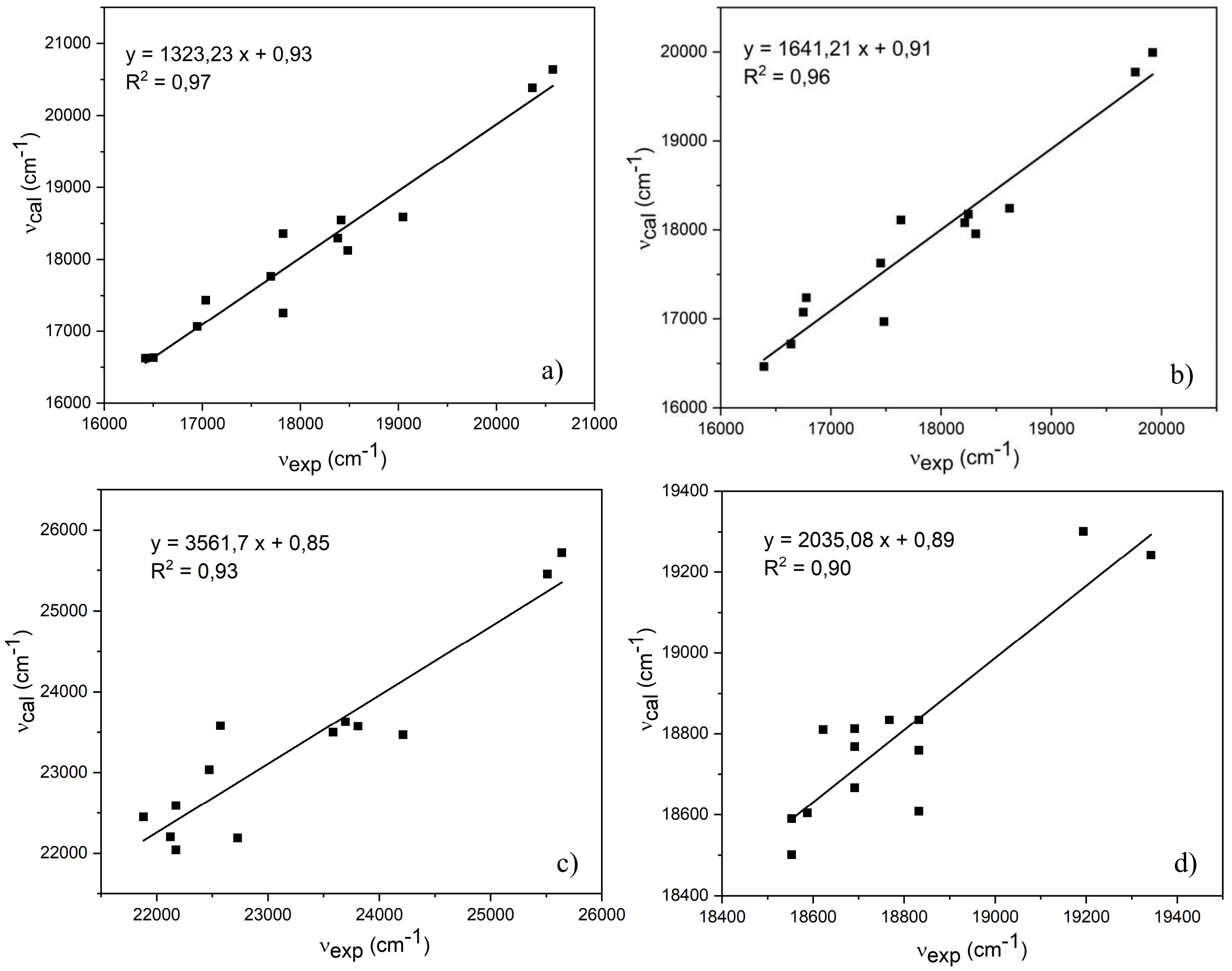

Figure S10. Representation of the calculated emission frequency (Kamlet–Taft) versus the experimentally determined one in the thirteen solvents for **1**, **2**, **3**, and $nSiO_2$-NHCO-CCM, corresponding to a), b), c), and d), respectively.

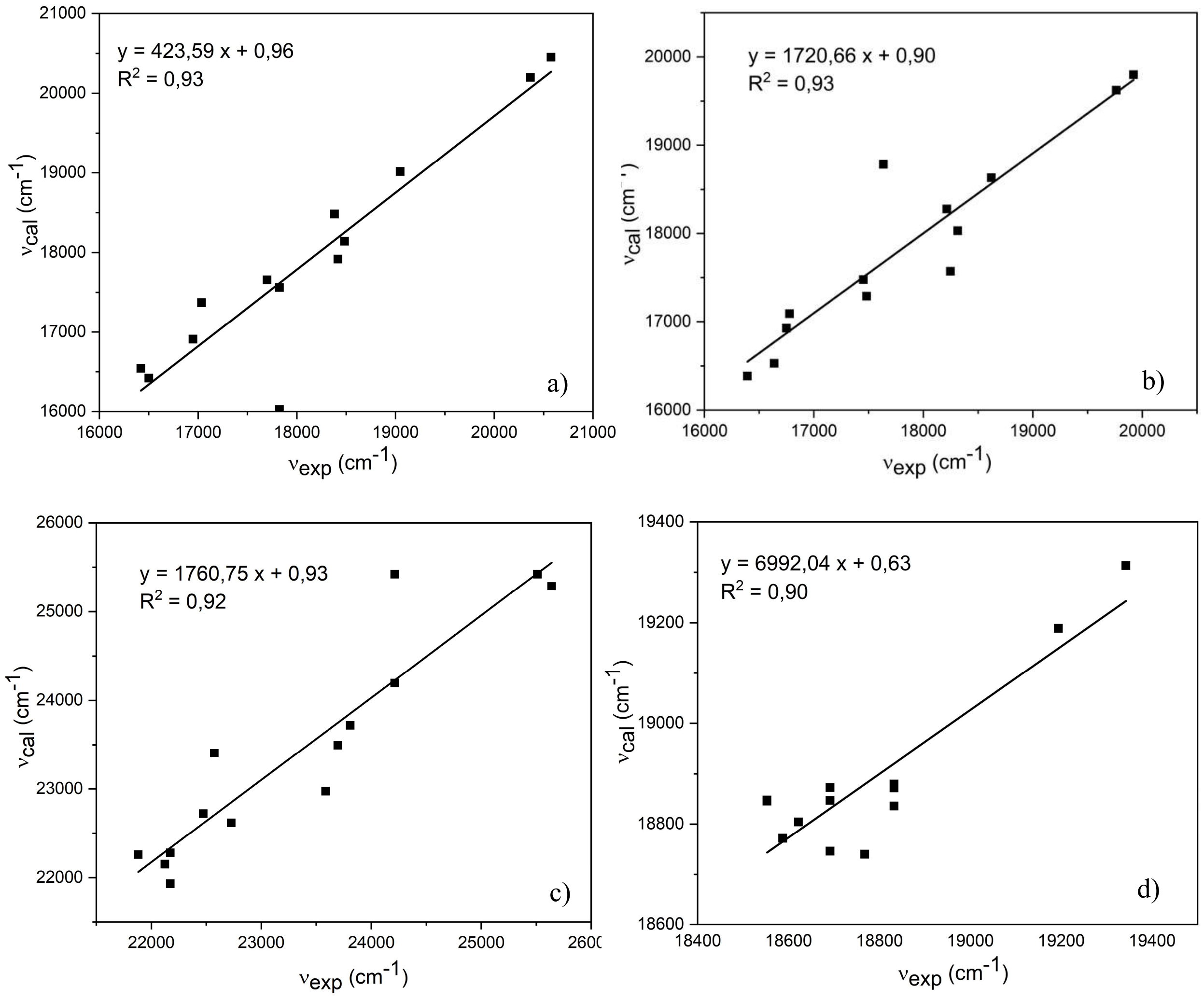

Figure S11. Representation of the calculated emission frequency (Catalán) versus the experimentally determined one in the thirteen solvents for **1**, **2**, **3**, and nSiO_2-NHCO-CCM, corresponding to a), b), c), and d), respectively.
